# Effect of Moisture Condition of Structural Lightweight Concretes on Specified Values of Static and Dynamic Modulus of Elasticity

**DOI:** 10.3390/ma16124299

**Published:** 2023-06-10

**Authors:** Lucyna Domagała, Kinga Sieja

**Affiliations:** Faculty of Civil Engineering, Cracow University of Technology, 31-155 Kraków, Poland; kingasieja@gmail.com

**Keywords:** lightweight concrete, modulus of elasticity, dynamic modulus of elasticity, secant modulus of elasticity, ultrasonic pulse velocity, compressive strength, density, moisture content

## Abstract

The dynamic modulus of elasticity (*E_d_*), specified by ultrasonic pulse velocity measurements, is often used, especially for concrete built into construction, to estimate the static modulus of elasticity (*E_c,s_*). However, the most commonly used Equations for such estimations do not take into account the influence of concrete moisture. The aim of this paper was to establish this influence for two series of structural lightweight aggregate concrete (LWAC) varying in their strength (40.2 and 54.3 MPa) and density (1690 and 1780 kg/m^3^). The effect of LWAC moisture content turned out to be much more pronounced in the case of dynamic modulus measurements than for static ones. The achieved results indicate that the moisture content of the concrete should be taken into consideration in modulus measurements as well as in Equations estimating *E_c,s_* on the basis of *E_d_* specified by the ultrasonic pulse velocity method. The static modulus of LWACs was lower on average by 11 and 24% in relation to dynamic modulus, respectively when measured in air-dried and water-saturated conditions. The influence of LWAC moisture content on the relationship between specified static and dynamic moduli was not affected by the type of tested lightweight concrete.

## 1. Introduction

The modulus of elasticity is one of the most important mechanical properties of structural concrete in the designing process for concrete construction. The knowledge of a value of this characteristic is necessary to determine the deflection of a structural member, stress–strain relation, losses of prestress force, crack width as well as stresses induced by strains associated with environmental effects. The values of modulus of elasticity given in the European Standard for designing concrete structures EN-1992-1-1 [1] should be regarded as indicative for general applications. However, the modulus of elasticity should be specifically determined if a structural element is likely to be sensitive to deviations from these standard values. 

A specified value of modulus of elasticity is affected by numerous material and technological parameters such as type and content of concrete constituent materials, concrete condition and method of testing. Nevertheless, it is the type of aggregate which determines the modulus especially strongly [2,3,4]. That is why the relationship between the modulus and compressive strength of concrete is not sufficient without taking into consideration the aggregate type [5,6]. In the case of normal-weight concrete (NWAC), EN-1992-1-1 [1] considers corrections of a value of modulus of elasticity in terms of aggregate type. For lightweight aggregate concrete (LWAC), the standard gives the possibility to estimate the value of the modulus based on its compressive strength and density. Such an approach indirectly takes into consideration the specificity of lightweight aggregate (LWA) and its content in concrete. This is particularly important due to the fact that in the case of structural lightweight concretes, the content of lightweight aggregate is a parameter through which the modeling of the properties of concrete composites is deliberately influenced. As a result, the estimated value of modulus of elasticity for LWAC may be lower by 15 up to 60% in comparison to NWAC of comparable strength class [1,7,8,9]. 

A parameter that can significantly affect the value of the modulus of elasticity, but which is rarely taken into account, is concrete moisture content. However, unlike many studies on the effect of concrete moisture content on its compressive strength (e.g., [2,10,11]), the effect on the modulus of elasticity is relatively rarely researched. Despite the fact that EN 12390-13 [12] specifies that specimens to be tested for modulus of elasticity should be in water-saturated conditions, in practice many tests are carried out on concrete in a different moisture condition. In particular, this applies to the cases of determining the modulus on specimens taken from a structure or non-destructive testing using the ultrasonic method. Meanwhile, as opposed to compressive strength, when the moisture content of concrete exceeds a certain level in relation to its saturation condition, the modulus of elasticity increases [2,9,13]. As a result, depending on the composition of concrete and its porosity structure, static modulus of elasticity tested in water-saturated conditions was observed as higher by 3 up to 55% in relation to dry conditions [9,14,15]. The increase is explained by the lower deformability of the material in which the pores are filled with water. On the other hand, in [16], it was revealed that concrete modulus generally decreased during drying due to cracks opening caused by differences in the change in volume between the aggregate and mortar. Therefore, concrete with stiffer and larger aggregates showed a bigger decrease in the modulus. There is also some research indicating that concrete strength and modulus of elasticity are inversely related to moisture content in the concrete (e.g., [13,17]). However, it should be stated that these studies mostly concern testing modulus in higher than standard temperatures and not by standard methods. Due to the fact that lightweight aggregate concrete is characterized by a significantly greater ability to accumulate water, the influence of concrete moisture content on the value of the modulus of elasticity may be more visible. Unfortunately, there is only a few research studies dealing with this problem. In [9], it was shown that for structural lightweight concrete the modulus determined in water-saturated conditions was higher by up to 14% than this specified in air-dry conditions (the degree of saturation ranged from 60 and 100%). The increase in modulus was more pronounced in the case of LWAC with higher water absorption. 

Since the stress–strain relationship for structural concrete under compressive load is usually curvilinear, there are several types of moduli of elasticity:

- tangent initial modulus specified at stress equaled to 0 (*E_0_* [1]),
- secant initial modulus determined at the first cycle (*E_c,0_* [12]);
- secant stabilized modulus determined after the third cycle (*E_c,s_* [12]).
- Besides the static moduli, mentioned above, a dynamic modulus (*E_d_*) is also specified for concrete. It is carried out in non-destructive tests causing vibrations in concrete, which result in stresses being vanishingly small. For this reason, the value of the dynamic and static initial moduli are often equated [2] and their values are bigger than the static secant modulus, which is the basic parameter used for designing the process of a concrete structure.

There are many relationships between static and dynamic moduli [2,18]. The most popular and the simplest is Equation (1) proposed by Lydon and Balendran [2] to determine static secant modulus on the basis of a value of dynamic modulus specified by measurements of ultrasonic pulse velocity.

(1) $$E_{c,s}=0.83\;E_{d}$$

Equations (2) and (3) describing relationships between *E_c,s_* and *E_d_*, dedicated also to LWAC, were proposed by Swamy and Popovics, respectively [2]. In Equation (3), additionally concrete density (*ρ*) and a constant depending on the unit system were taken into consideration (*k*). Nevertheless, parameter *k* was specified for units in psi (0.23), while in SI is unidentified [18].

(2) $$E_{c,s}=1.04\;E_{d}-4.1$$

(3) $$E_{c,s}=k\;{E_{d}^{1.4}\rho^{-1}}_{}$$

The above Equations, especially (1), are often used to estimate the static secant modulus of concrete built into a construction instead of its direct testing on specimens cored from the structure. Meanwhile, some scientific papers, in which the results of both types of moduli were tested, indicate that the relationship between *E_c,s_* and *E_d_* may be significantly different from estimations given by the proposed relationships [19,20,21,22,23,24,25,26]. For example, the ratio of static and dynamic moduli of normal-weight concretes determined in water-saturated conditions, calculated on the basis of data given in [26], ranged from 0.72 to 0.76. Other concretes of the same compositions but cured in air and tested in air-dried conditions showed visibly higher ratios of 0.79–0.87. As a result, Equation (1) gives an overestimated value of static modulus on average by 10% for concretes cured in water and tested in saturated conditions, while it seems to be accurate enough for concretes cured in air and tested in air-dry conditions. In the first case, the estimated values were higher by 2.5–3.5 GPa, while in the second case, the differences were up to 0.6 GPa. Verification of Equation (2) gives much bigger differences in estimation. The calculated value of static modulus was higher in relation to the measured value on average by 25 and 10%, respectively for concretes cured in water and cured in air. As a result, the values of static modulus were overestimated even by up to 9 GPa. The results given in [22,23] proved that both relationships overestimate the values of static modulus in comparison to the measured values and Equation (2) gives much higher results. The estimated *E_c,s_* was higher by up 50% and the difference in overestimation of the static modulus was up to 15 GPa. In turn, the analysis of results for structural lightweight concretes, presented in [24,25], shows much lower differences in measured values of dynamic and static moduli (0 to 4 GPa) than for NWACs reported in [22,23,26] (4 to 17 GPa). Therefore, for the reported LWACs, the above-mentioned relations underestimate a value of *E_c,s_*. by up to 4.5 GPa (on average by 12%) and 3.0 GPa (on average by 8%), respectively when applied Equations (1) and (2). It should be noted that the difference between measured and estimated values of static modulus of elasticity was higher when the initial moisture content of the lightweight aggregate was bigger. Such an observation may be explained by the different microstructure of LWACs with aggregates of different initial moisture content. As it was proved in [9,27], the less initial moisture content of LWA, the better adhesion between cement paste and the aggregate and the more linear stress–strain relationship.

According to [28], the pulse velocity is strongly influenced by not only the average moisture content but also its distribution. The length of the crack and the degree of filling with liquid is also important. Although it is assumed that the relationships between *E_c,s_* and *E_d_* are independent of the method of curing, air entrainment, cement type or test condition [2,18], the results presented in [26] indicate that the relationships between dynamic and static moduli may be dependent on the concrete moisture condition. Nevertheless, this thesis should be proved in research, as concrete reported in [26], although had the same compositions, in fact, had considerably different microstructure and mechanical properties due to different curing conditions. 

In the case of lightweight concretes, the effect of moisture content on the specified values of the static and dynamic modulus may be different than in the case of NWAC. On the one hand, since LWAC usually reveals much higher water absorption than normal-weight concrete, it may be expected to show an even much more pronounced influence of moisture content on the modulus of elasticity. On the other hand, since LWAC, in comparison to NWAC, is characterized usually by more homogeneous structure and therefore more linear stress–strain relation, the difference between values of *E_c,s_* and *E_d_* may be less considerable than for normal-weight concrete.

The main aim of the research was to check the influence of lightweight concrete moisture content on specified values of modulus of elasticity determined as dynamic one in non-destructive ultrasonic pulse velocity method as well as static secant one determined in cycles of compressive loading and unloading. The additional purpose was to verify relationships between these two types of moduli.

## 2. Materials and Methods

The tests were carried out on standard cylindrical specimens cast of two series of structural lightweight aggregate concretes characterized by different strengths and densities. The various properties of these two LWACs were achieved by different nominal water and cement ratios of cement matrix (*w/c*): 0.37 and 0.55. To ensure similar workability of both fresh concretes, a superplasticizer was applied for LWAC with lower *w/c*. However, both concretes were made of Portland cement CEM I 42.5 R, potable water, natural sand 0/2 mm as fine aggregate and sintered fly ash aggregate 4/8 mm (Lytag) as coarse aggregate. Both series were characterized by the same volume proportions between cement matrix (mortar) and lightweight aggregate (55%:45%). 

**Figure 1:**
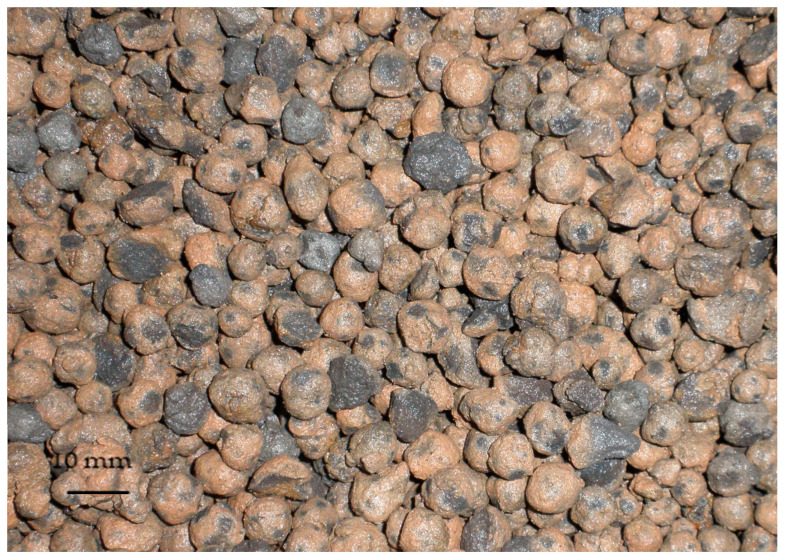
Sintered fly ash aggregate 4/8 mm (Lytag) used for concretes preparation.

The basic properties of the used lightweight aggregate fraction, presented in Figure 1, were the following: the crushing resistance, tested according to EN 13055-1 [29], 8.0 MPa; the particle density, specified in accordance with EN 1097-6 [30], 1320 kg/m^3^; the bulk density tested in accordance to EN 1097-6 [31], 730 kg/m^3^; the water absorption, determined according to EN 1097-6 [30] after immersion in water for 24 and 72 h reached 18.8 and 25.3%, respectively. Analyzing the above properties of the lightweight aggregate and comparing them to properties of other lightweight aggregates [2,32,33,34,35,36], it should be stated that despite rather high-water absorption, it reveals relatively high crushing resistance which made this LWA one of the most suitable for structural concretes. 

To minimize the risk of fresh concrete workability loss the lightweight aggregate was initially wetted to the moisture content of 17.0% by mass which corresponded to LWA water absorption after immersion in water for 1 h. The compositions of prepared fresh concretes are presented in Table 1.

**Figure 2:**
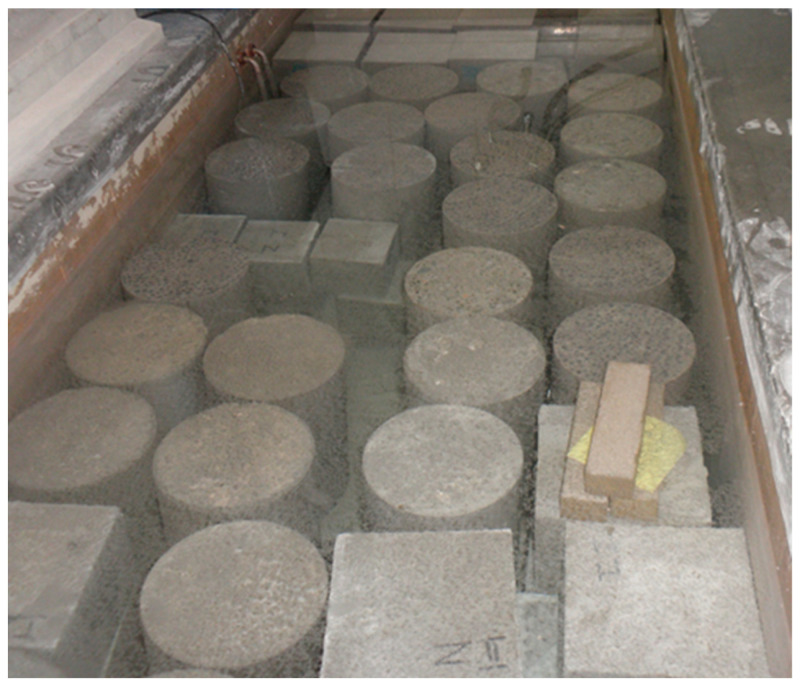
Specimens cured for tests in saturated condition.

For each concrete, series 21 standard cylinders (150/300 mm) were molded and compacted on a vibration table. All 42 specimens were demolded after 24 h and cured in a climatic chamber (RH = 100%, T = 20 °C) according to EN 12390-2 [37] for 28 days. Figure 2 the condition of the specimens curing. 

The concretes were tested at two ages: 28 days and 3 years. At the first age, the concrete density and strength were determined to classify both concretes. The actual tests of dynamic and static moduli of elasticity were carried out at the age of 3 years. This late age of the research ensured that the time of testing had no effect on the specified values of the modulus of elasticity. The tests carried out, the LWACs specimens’ number and age at testing, as well as the standard test procedures for each concrete series are given in Table 2.

The tests of compressive strength and modulus of elasticity at 28 days were carried out on specimens in saturated conditions as it is required by the European Standards EN 12390-3 [39] and EN 12390-13 [12]. The density at 28 days was determined firstly in saturated condition and then in oven-dried condition to determine both water absorption and density class. Meanwhile, the specimens to be tested at the age of 3 years were removed from the climatic chamber after 28 days of curing in water and stored in a laboratory room (RH = 50%, T = 20 °C) until the date of the tests. Therefore, at a later age the tests of dynamic and static moduli of elasticity were carried out on specimens in air-dry (as-received) condition first. Then, the specimens were saturated in water for two months to the constant mass and in this condition, they were tested once again. The compressive strength and density were tested at this late age in both conditions: air-dry and saturated.

**Figure 3:**
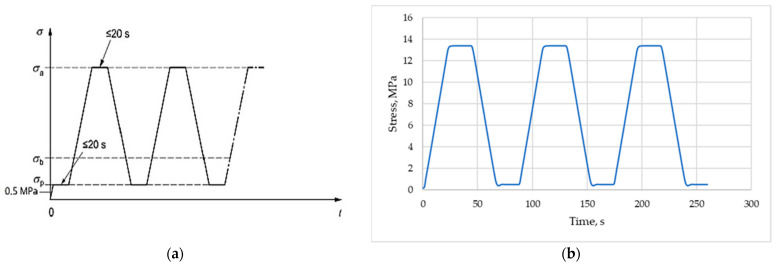
Load cycles for determination on secant modulus of elasticity: (**a**) Theoretical cycles acc. to EN 12390-13 (Method B); (**b**) Example of registered carried out load cycles for a specimen of concrete series LC1.

At a given moisture condition, the static secant modulus of elasticity (*E_c,s_*) was tested according to Method B of the standard EN 12390-13 [12] firstly and then the dynamic modulus (*E_d_*) was specified using ultrasonic pulse velocity method as described in EN 12504-4 [40]. Therefore, to determine the loading and unloading range for testing the secant modulus, 3 cylinders of each concrete series were subjected to compressive strength test at the beginning. As a result, for each concrete series, the mean value of compressive strength (*f_cm, cyl_*) was calculated. Finally, the nominal lower stress (*σ_p_*) and the nominal upper stress (*σ_a_*) were assumed as 0.5 MPa and *f_cm, cyl_*/3, respectively. The standard scheme of loading and unloading cycles, as well as an example of cycles registered at testing, are presented in Figure 3. The testing procedure according to Method B consists of three loading cycles in the stress range from the lower stress *σ_p_* up to the upper stress *σ_a._* The stabilized secant modulus of elasticity was determined according to Equation (4), where *σ_a_^m^* and *σ_b_^m^* are stress values measured at the third cycle, while *ε_a,3_* and *ε_p,2_* are corresponding strains.

(4) $$E_{c,s}=\frac{\sigma_{a}^{m}-\sigma_{p}^{m}}{\varepsilon_{a,3}-\varepsilon_{p,2}}$$

The dynamic modulus of elasticity was estimated on the basis of Equation (5) adopted from ASTM C 215 [18], where *V* is a measured velocity of ultrasonic wave; *D* is a specified concrete apparent density and *ν* is the concrete Poisson’s ratio, assumed as 0.2.

(5) $$E_{d}=\frac{V^{2}D(1+\nu )(1-2\nu )}{\left(1-\nu \right)}$$

**Figure 4:**
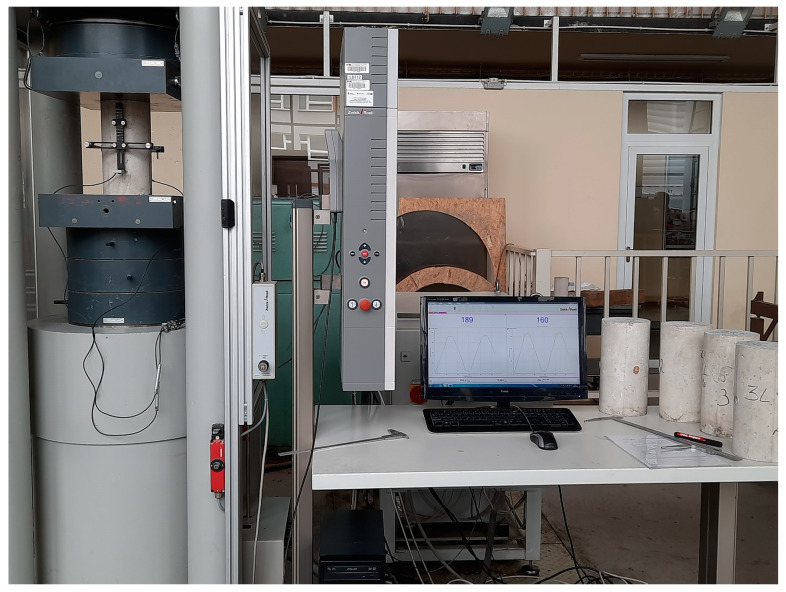
A concrete specimen with the measuring apparatus for determination of secant modulus of elasticity according to EN 12390-13.

**Figure 5:**
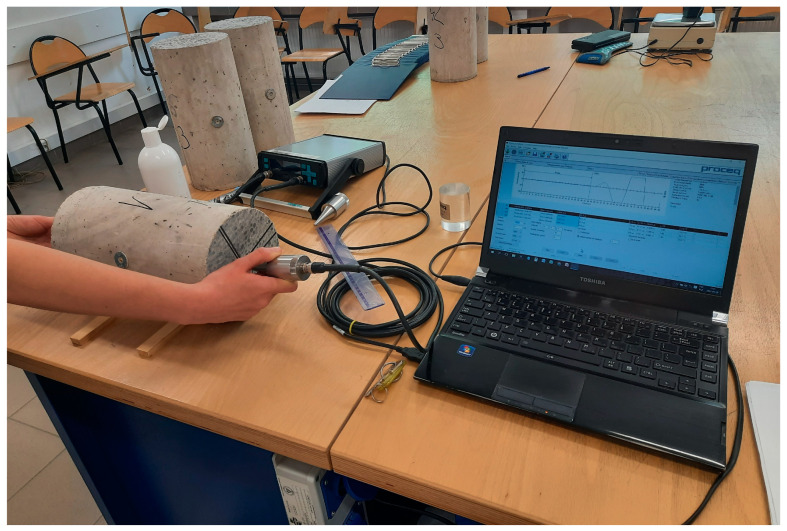
A concrete specimen with the measuring apparatus for determination of dynamic modulus of elasticity according to ultrasonic pulse velocity method.

Since EN 12504-4 [40] assumed that ultrasonic test instruments can indicate a tendency for velocity to reduce slightly with increasing path length due to the heterogeneous nature of concrete, the probes were located in three positions: once on the opposite bases of cylindrical specimens along their height (path length ≈ 300 mm) and twice on the opposite cylinders sidewalls in two perpendicular directions (path length ≈ 150 mm). In each position of the probes, about 30 measurements of pulse velocity were carried out and the mean value was determined. The measuring positions with equipment for determination of secant and dynamic modulus of elasticity are presented in Figure 4 and Figure 5, respectively. 

To eliminate the influence of concrete heterogeneity on the result of static and dynamic modulus measurements, both tests were carried out in sequences on the same set of specimens including both moisture conditions. Such an approach was assumed reliable owing to the relatively low upper stress considered in multiple cyclic loading (*f_cm, cyl_*/3) that is significantly lower than the fatigue strength. As was stated in [2,10,11], cyclic loading under the fatigue strength should not affect a test result. Such behavior of tested lightweight concretes under cyclic load was also proved in this research. One selected specimen of each concrete series was subjected three times to tests of static modulus of elasticity in different arrangements of sensors in relation to a specimen and a specimen in relation to the machine platens. The measurement repeatability was satisfactory and showed no visible effect of multiple cyclic loading on a specified result of the test.

## 3. Results

### 3.1. Results of Preliminary Tests at the Standard Age of 28 Days

The average compressive strength determined on cylindrical specimens in water-saturated conditions at 28 days was 33.5 and 45.3 MPa, respectively for series LC1 and LC2. The corresponding standard deviations were 0.6 and 1.6 MPa.

The average static modulus of elasticity determined on cylindrical specimens in water-saturated conditions at 28 days was 17.5 and 21.6 GPa, respectively for series LC1 and LC2. The standard deviation of the modulus of elasticity was 0.2 GPa in the case of both concrete series. 

The average density under the water-saturated condition at 28 days was 1840 and 1890 kg/m^3^, respectively for series LC1 and LC2. Meanwhile, the corresponding average oven-dried density was 1680 and 1770 kg/m^3^. Any individual result did not differ from the mean value of more than 20 kg/m^3^. 

### 3.2. Results of Main Tests at the Age of Three Years

The average compressive strength determined on cylindrical specimens in water-saturated conditions at the age of three years was 40.2 and 54.3 MPa, respectively for series LC1 and LC2. The corresponding standard deviations were 1.2 and 1.3 MPa. 

The average density under air-dried conditions (as received) at the age of three years was 1710 and 1810 kg/m^3^, respectively for series LC1 and LC2. Meanwhile, the corresponding average density under the water-saturated conditions were 1860 and 1920 kg/m^3^, and the corresponding average oven-dried density was 1690 and 1780 kg/m^3^. Any individual result did not differ from the mean value more than 20 kg/m^3^. 

The water absorption, calculated on the basis of concrete density in water-saturated and oven-dried conditions, was 10.1 and 7.9%, respectively for LC1 and LC2. The moisture content in concrete to be tested in air-dried conditions at the age of three years was 1.2 and 1.7%, respectively for LC1 and LC2.

**Figure 6:**
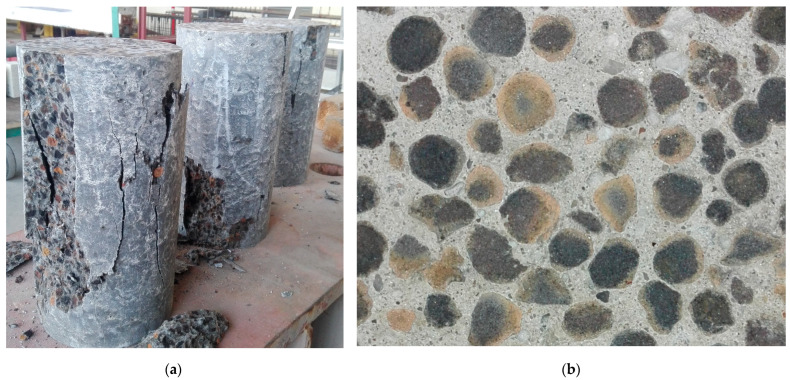
Specimens of lightweight concrete after compressive strength test: (**a**) General view; (**b**) Fracture form.

The failure and fracture form of cylindrical specimens at compressive strength tests showed good structural homogeneity of both tested concretes (Figure 6). There was no sign of concrete segregation and, despite the initial saturation of LWA, the adhesion between the cement matrix and lightweight aggregate turned out to be enough strong so that the cracks did not pass through the bond of any specimen.

#### 3.2.1. Static Secant Modulus of Elasticity

According to achieved results of compressive strength (see Section 3.2), the upper stress in loading cycles for determination of static secant modulus of elasticity was assumed as 13.4 and 18.1 MPa, respectively for series LC1 and LC2.

The specified values of secant modulus of elasticity tested in two conditions: air dried and water saturated are presented in Table 3. The mean modulus of elasticity determined in air-dried conditions was 18.3 and 21.9 GPa, respectively for series LC1 and LC2. While in water-saturated conditions, the corresponding values were: 18.4 and 22.8 GPa. Standard deviations of the specified modulus ranged from 0.1 to 0.6 GPa.

#### 3.2.2. Dynamic Modulus of Elasticity

The values of ultrasonic pulse velocity, measured in two conditions: air dried and water saturated, are presented in Table 4. The mean velocity determined in the air-dried condition was 3706 and 3904 m/s, respectively for series LC1 and LC2. While in water-saturated conditions, the corresponding values were 4042 and 4259 m/s. Standard deviations of specified velocity ranged from 23 to 52 m/s.

The values of dynamic modulus of elasticity, calculated on the basis of measured ultrasonic pulse velocity using Equation (5), for both conditions: air dried and water saturated are presented in Table 5. The mean modulus of elasticity determined in air-dried conditions was 20.9 and 24.4 GPa, respectively for series LC1 and LC2. While in water-saturated conditions, the corresponding values were: 24.9 and 29.1 GPa. Standard deviations of specified modulus ranged from 0.3 to 0.6 GPa.

**Figure 7:**
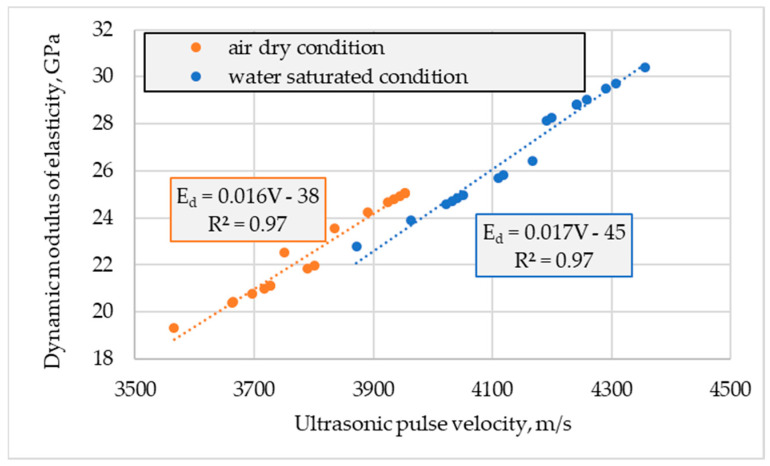
Relationship between ultrasonic pulse velocity (*V*) and dynamic (*E_d_*) moduli of elasticity is determined in air-dried and water saturated conditions.

The relationship between measured values of ultrasonic pulse velocity and values of dynamic modulus of elasticity, specified on these measurements, are shown in Figure 7. It is clearly visible that this relationship is dependent on both: the type of concrete considered in Equation (5) by its density and by the moisture condition. The influence of the last factor seems to be stronger. Nevertheless, it is not taken into account in the Equation.

## 4. Discussion

As it was assumed, various water-cement ratios ensured significantly different levels of compressive strength, modulus of elasticity and density for both concrete series. As a result, in accordance with the standard EN-206 [41], concrete LC1 may be classified as the strength class LC 25/28, while LC2 is LC40/44. Nevertheless, despite the visible difference in density, the density of both concretes is classified as D1.8.

At the age of three years, both series revealed pronounced strength development. The mean value of compressive strength at this age time was higher by 20% in relation to the strength determined at the standard age of 28 days. Such an increment in strength should be assessed as relatively high as for structural concretes [34,42,43]. It was probably possible due to internal curing with a significant amount of water accumulated in lightweight aggregate characterized by its high-water absorption. The corresponding development of the secant modulus of elasticity during three years was not so pronounced. Both concretes revealed an increment of the modulus tested in water-saturated conditions of only about 6%. Although the specified modulus values are lower than the standard estimations according to EN 1992 [1], the modulus development rate in time is consistent with the data given in the standard. 

**Figure 8:**
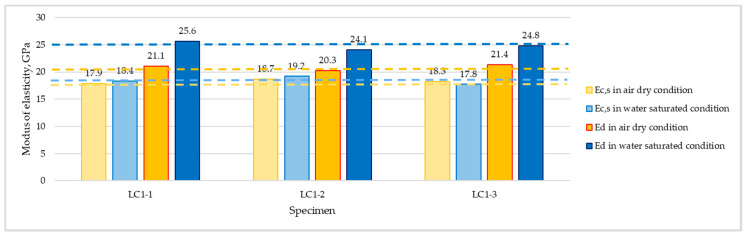
Values of static secant (*E_c,s_*) and dynamic (*E_d_*) moduli of elasticity determined for concrete LC1 in air-dried and water-saturated conditions.

**Figure 9:**
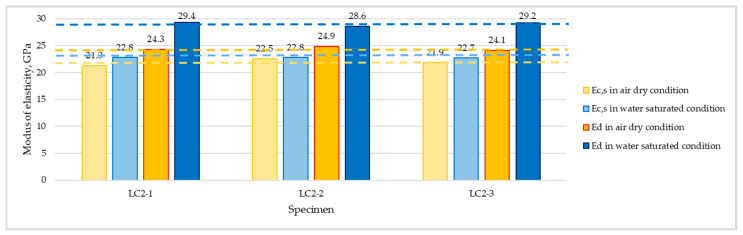
Values of static secant (*E_c,s_*) and dynamic (*E_d_*) moduli of elasticity determined for concrete LC2 in air-dried and water-saturated conditions.

The comparison of specified values of static secant and dynamic moduli tested in both considered moisture conditions for series LC1 and LC2 are presented in Figure 8 and Figure 9, respectively. The values of dynamic modulus of elasticity for individual specimens were calculated as an average of one longitudinal and two transverse measurements. Although the standard EN 12504-4 [40] assumed that ultrasonic test instruments can indicate a tendency for velocity to reduce slightly with increasing path length, in the case of both tested concretes there was no visible effect of path length (150 or 300 mm) on the determined ultrasonic pulse velocity. 

### 4.1. Relationship between Static Secant and Dynamic Moduli of Elasticity

**Figure 10:**
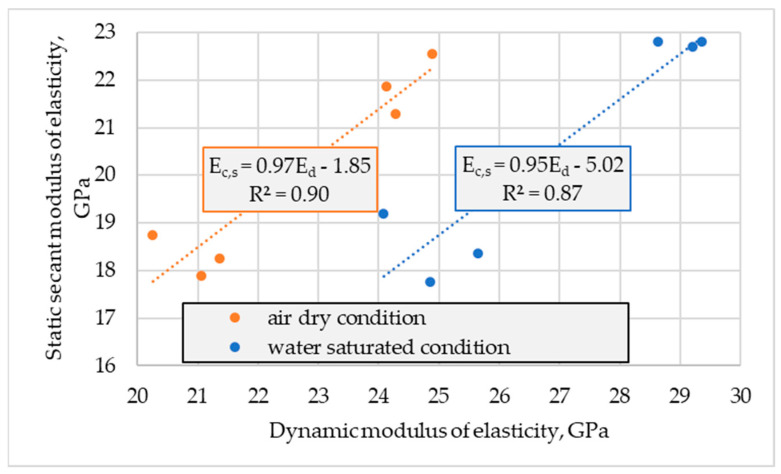
Relationship between static secant (*E_c,s_*) and dynamic (*E_d_*) moduli of elasticity determined in air-dried and water saturated conditions.

The carried out tests proved considerably higher values of dynamic modulus of elasticity in relation to static secant values, regardless of concrete series and the moisture content of specimens (Figure 10). 

The ratio of *E_c,s_* and *E_d_* determined for individual specimens tested in air-dried conditions ranged from 0.85 to 0.93, while specified in water-saturated conditions ranged from 0.71 to 0.80. The effect of the concrete series was not observed. As a result, the average ratio *E_c,s_* / *E_d_* was assessed as 0.89 and 0.76 for air-dried conditions and water-saturated conditions, respectively. In both cases, the standard deviation of the ratio was 0.03. The obtained ratio values for tested lightweight concretes were slightly lower than those calculated for LWACs reported in [24,25]. On the other hand, the obtained *E_c,s_* / *E_d_* turned out to be insignificantly higher than those calculated on the basis of data given in [26] for normal-weight concretes and much higher when compared to those calculated for NWACs reported in [22,23]. As in the case of [26], in this research, the value of *E_c,s_* / *E_d_* determined in air-dried conditions was considerably higher than for water-saturated concretes. The above comparison of ratios achieved and calculated on the basis of reported data indicates that *E_c,s_* / *E_d_* is affected by both moisture content and the homogeneity of the concrete composite structure. The less homogeneous structure of a composite (the weaker bond between cement paste and aggregate or the bigger difference in stiffness of these two constituents materials or the bigger extent of concrete degradation due to detrimental exposure), the lower the ratio.

The obtained results showed that the application of Equation (1) or (2) to determine the secant modulus of elasticity of tested lightweight concretes in standard water-saturated conditions would lead to overestimation on average by 9 and 16%, respectively. For lightweight concretes tested in standard water-saturated conditions, Equation (6) should be proposed as more reliable:

(6) $$E_{c,s}=0.76\;E_{d}$$

In turn, for LWACs in air-dried conditions, both Equations (1) and (2) slightly underestimate the value of *E_c,s_*, on average by 6 and 3%, respectively. 

If the values of the secant modulus of elasticity of concretes in a saturated condition, as meeting the requirements of the standard EN 12390-13 [12], were estimated according to Equations (1) and (2) on the basis of dynamic modulus measured on concrete in air-dried conditions, as it is in construction, the value would be underestimated by 8% and 5%, respectively.

It should be noted that since there were no visible differences in the relationship between the specified moduli for both tested concretes, Equation (3) taking into consideration concrete density, would not be appropriate in this case.

### 4.2. The Influence of Moisture Content on Specified Values of Modulus of Elasticity

**Figure 11:**
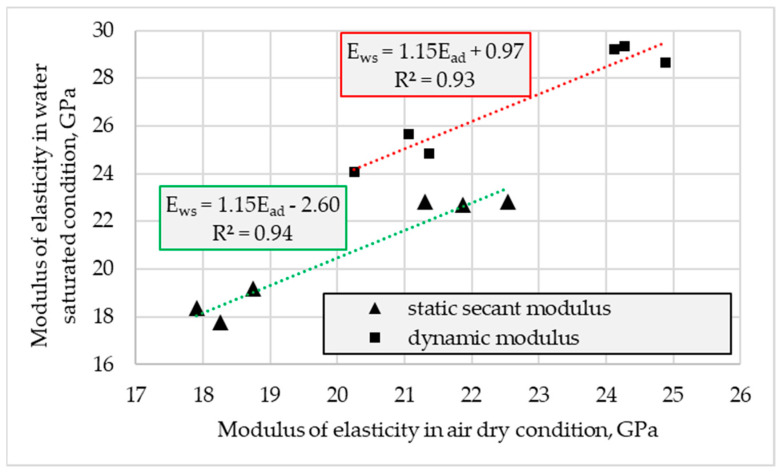
The effect of moisture condition on specified values of static secant and dynamic moduli of elasticity determined in air-dried conditions (*E_ad_*) and water-saturated conditions (*E_ws_*).

The carried out tests proved the effect of concrete moisture content on the specified values of both tested moduli (Figure 11). The values of static secant and dynamic moduli determined in water-saturated conditions were higher than those specified in air-dried conditions. However, in the case of determination of the static secant modulus, the influence of moisture content turned out to be much less pronounced than for the dynamic modulus. The values of *E_c,s_* determined in water-saturated conditions were higher than the values tested in the air-dried conditions by only 2% on average. Meanwhile, for the dynamic modulus, such a mean increment was as high as 19%. No visible influence of the concrete series was observed.

The influence of the moisture content of the tested concretes on the values of the static secant modulus of elasticity turned out to be less significant than in the case of the studies discussed in [9,13,14,15]. As it was shown in [13,14,15], the typical increase in *E_c,_*_s_ of normal-weight concrete tested in water saturated condition ranged from 12 to 32% in relation to the dry condition. It should be noted that although the referred concretes had similar strength to those tested in this research, the porosity structure of composites was different. To achieve a similar strength level of concrete made of a more porous aggregate, it is necessary to use cement paste tighter than in the case of normal-weight concrete. Therefore, in saturated condition there is less water in the cement matrix of LWAC than of NWAC. Moreover, the bond between aggregate and cement paste in LWAC is usually better. However, when comparing the results achieved in this research to those referred to in [9] for lightweight concrete, a certain quantitative difference is also observed for the effect of moisture content on the static modulus of elasticity. As it was revealed in [9], *E_c,s_* determined in water-saturated conditions was bigger by 4 up to 14% in relation to results measured in air-dried condition. In this case, the explanation of a more pronounced moisture influence on the modulus in [9] is the much higher moisture content of lightweight concretes in air-dried conditions (5.7–7.8%). Meanwhile, in this research, due to longer time of drying in air (almost three years), LWACs tested in dry conditions had a relatively small moisture content (1.2–1.7%). As a result, in [9], air-dried conditions meant the saturation extent was 62–73%, and in this study, it ranged from 12 to 22%. Such a comparison may prove the conclusions given in [13] that static modulus of elasticity may decrease with increasing the moisture content to a certain level of concrete saturation with water (50–70%) and then may increase up to the full saturation.

## 5. Conclusions

The tests and the analyses of obtained results proved that lightweight concrete tested in standard water-saturated conditions revealed higher values of both specified moduli, static and dynamic, in relation to measurements achieved in air-dried conditions. However, the influence of the LWAC moisture content turned out to be much more pronounced in the case of dynamic modulus measurements. Such an observation indicates that the moisture content of the concrete should be taken into consideration in modulus tests as well as in Equations estimating the static secant modulus of elasticity on the basis of ultrasonic pulse velocity measurements.

For tested concretes, the static modulus of elasticity was lower on average by 11 and 24% in relation to the dynamic modulus, respectively for lightweight concretes tested in air-dried and water-saturated conditions. Therefore, in the case of tested LWACs, the commonly used relationships between static and dynamic moduli ((1) and (2)) turned out to be more accurate than for some normal-weight concretes. Nevertheless, even for the tested lightweight concretes the Equations also required correction. 

The influence of LWAC moisture content on the specified static and dynamic module was not affected by the type of tested lightweight concrete, determined by its various strength and density level. However, the comparison of results achieved in this research and calculated on the basis of reported data indicates that the relationship between *E_c,s_* and *E_d_* is influenced by both moisture content and the homogeneity of concrete composite structure. As a result, the tested lightweight concrete, despite their higher water absorption in comparison to NWACs, did not show a bigger effect of their moisture content on the specified values of static modulus of elasticity, probably due to better LWACs’ structural homogeneity and its more tight cement matrix. Nevertheless, it should be stated that the influence of the moisture content of concrete on its modulus of elasticity is not as unequivocal as in the case of compressive strength tests.

In order to establish a reliable and versatile relationship between the static and dynamic moduli of elasticity for structural concretes, further tests should be carried out on different types of cement composites in a wider range of their moisture content.

## Figures and Tables

**Table 1:** Composition of lightweight concretes (LC) to be tested in kg/m^3^.

Series	LWA 4/8 mm in Dry Condition	Water for Initial Wetting of LWA	Natural Sand 0/2 mm	Cement	Water	Superplasticizor
LC1	576	98	570	377	208	0
LC2	576	98	570	470	174	1.9

**Table 2:** Test type; cylindrical specimens’ number and age; standard procedure for each concrete series.

Test	Specimens Number	Concrete Age	Standard Procedure
Water saturated density	3	28 days	EN 12390-7 [38]
Oven dry density			
As received density			
Oven dry density	3	3 years	
Water saturated density			
Compressive Strength	6	28 days	EN 12390-3 [39]
	3	3 years	
Static modulus of elasticity	3	28 days	EN 12390-13 [12]
Static modulus of elasticity	3	3 years	EN 12390-13 [12]
Dynamic modulus of elasticity			EN 12504-4 [40]

**Table 3:** Results of measurements of static secant modulus of elasticity.

Concrete Series	Specimen’ Number	Secant Modulus of Elasticity, GPa				
		Air Dry Condition			Water Saturated Condition	
		*E_c,si_* *	*E_c,si_*	*E_c,s_*	*E_c,si_*	*E_c,s_*
LC1	1	18.1	17.9	18.3	18.4	18.4
	1’	17.9				
	1”	17.7				
	2	-	18.7		19.2	
	3	-	18.3		17.8	
LC2	1	21.4	21.3	21.9	22.8	22.8
	1’	21.7				
	1”	20.8				
	2	-	22.5		22.8	
	3	-	21.9		22.7	

* Measurements repeated on the same specimen: ’ first repetition; ” second repetition.

**Table 4:** Results of measurements of ultrasonic pulse velocity.

Concrete Series	Specimen’ Number	Measurement *	Path Length, mm	Ultrasonic Pulse Velocity, m/s					
				Air Dry Condition			Water Saturated Condition		
				*V_i_*	*V_m_*	*V*	*V_i_*	*V_m_*	*V*
LC1	1	L	295	3665			4032		
		T	151	3801	3721		4167	4106	
		T’	151	3697			4119		
	2	L	296	3565			4042		
		T	150	3663	3648	3706	3872	3979	4042
		T’	150	3716			4022		
	3	L	296	3727			4051		
		T	151	3789	3748		3963	4041	
		T’	151	3727			4109		
LC2	1	L	301	3890			4242		
		T	150	3953	3893		4242	4280	
		T’	150	3835			4357		
	2	L	298	3953			4291		
		T	150	3944	3941	3904	4191	4227	4259
		T’	150	3925			4200		
	3	L	296	3935			4242		
		T	151	3953	3880		4258	4269	
		T’	151	3751			4308		

* L—longitudinal measurement; T—transverse measurement.

**Table 5:** Results of measurements of dynamic modulus of elasticity.

Concrete Series	Specimen’ Number	Measurement *	Path Length, mm	Dynamic Modulus of Elasticity, GPa					
				Air Dry Condition			Water Saturated Condition		
				*E_di_*	*E_dm_*	*E_d_*	*E_di_*	*E_dm_*	*E_d_*
LC1	1	L	295	20.4			24.7		
		T	151	22.0	21.1		26.4	25.6	
		T’	151	20.8			25.8		
	2	L	296	19.3			24.8		
		T	150	20.4	20.3	20.9	22.8	24.1	24.9
		T’	150	21.0			24.6		
	3	L	296	21.1			25.0		
		T	151	21.8	21.4		23.9	24.8	
		T’	151	21.1			25.7		
LC2	1	L	301	24.2			28.8		
		T	150	25.0	24.3		28.8	29.4	
		T’	150	23.6			30.4		
	2	L	298	25.0			29.5		
		T	150	24.9	24.9	24.4	28.1	28.6	29.1
		T’	150	24.7			28.3		
	3	L	296	24.8			28.8		
		T	151	25.0	24.1		29.0	29.2	
		T’	151	22.5			29.7		

* L—longitudinal measurement; T—transverse measurement.

## Data Availability

Not applicable.
